# Integrated heart rate and oxygen uptake recovery phenotypes reveal heterogeneous recovery patterns in coronary artery disease

**DOI:** 10.3389/fmed.2026.1894821

**Published:** 2026-07-09

**Authors:** Benil Nesli Ata, Baris Unal, Merve Celik, Bugra Ince, Ece Cinar

**Affiliations:** 1 Department of Physical Medicine and Rehabilitation, Izmir City Hospital, Bayrakli, Izmir, Türkiye; 2 Department of Cardiology, Izmir City Hospital, Bayrakli, Izmir, Türkiye; 3 Department of Physical Therapy, Izmir City Hospital, Bayrakli, Izmir, Türkiye; 4 Department of Physical Medicine and Rehabilitation, University of Health Sciences, Izmir City Hospital, Bayrakli, Izmir, Türkiye; 5 Department of Physical Medicine and Rehabilitation, Ege University School of Medicine, Bornova, Izmir, Türkiye

**Keywords:** cardiac rehabilitation, cardiopulmonary exercise testing, coronary artery disease, heart rate recovery, oxygen consumption, ventilatory efficiency

## Abstract

**Introduction:**

Heart rate recovery (HRR) and oxygen uptake (VO₂) recovery provide complementary information regarding autonomic and metabolic recovery following exercise; however, their integrated assessment remains insufficiently explored in patients with coronary artery disease (CAD).

**Methods:**

This retrospective observational study included 200 patients with stable CAD who underwent symptom-limited cardiopulmonary exercise testing (CPET). HRR at 1 min (HRR1), ΔVO₂ values, and VO₂ recovery indices were analyzed, and patients were classified according to combined HRR and VO₂ recovery patterns using a 2 × 2 recovery framework.

**Results:**

HRR1 was significantly correlated with ΔVO₂1 (*r* = 0.519, *p* < 0.001) and ΔVO₂2 (*r* = 0.510, *p* < 0.001), as well as VO₂ recovery indices at 1 and 2 min (*r* = 0.496 and 0.531, respectively; both *p* < 0.001). Distinct concordant and discordant recovery phenotypes were identified, indicating heterogeneity in post-exercise recovery dynamics. Significant differences were observed across recovery groups, including significantly lower VO₂ recovery in the Good HRR–Poor VO₂ phenotype compared with the Poor HRR–Good VO₂ phenotype (*p* = 0.027).

**Discussion:**

Post-exercise recovery in CAD appears to be a multidimensional physiological process that cannot be fully characterized by a single parameter. Integrated assessment of HRR and VO₂ recovery may provide a framework for recovery characterization before cardiac rehabilitation, and prospective studies are needed to validate the clinical applicability of this approach.

## Introduction

Functional impairment is a central clinical feature of stable coronary artery disease (CAD) and a major determinant of exercise tolerance and prognosis (1–3). Cardiopulmonary exercise testing (CPET) is the gold standard for objective assessment of functional capacity, integrating cardiovascular, pulmonary, and metabolic responses to physiological stress (4). Peak oxygen uptake (VO₂peak), the most commonly reported CPET parameter, is strongly associated with mortality and adverse cardiovascular events in CAD populations (3). However, peak indices reflect only maximal performance and may not fully capture post-exercise physiological recovery. Parameters such as heart rate recovery (HRR) and oxygen uptake kinetics provide complementary insight into autonomic reactivation and metabolic restitution following exertion (5, 6).

Exercise recovery represents an active and prognostically relevant phase of cardiovascular response. Impaired heart rate recovery (HRR) has been consistently associated with increased cardiovascular mortality, while delayed VO₂ recovery indicates reduced aerobic efficiency and compromised hemodynamic reserve (7–9). Importantly, recovery-phase variables may provide prognostic information independent of peak exercise performance (10). Thus, assessment of post-exercise responses may offer clinically meaningful insight beyond maximal CPET indices.

Existing studies have predominantly evaluated heart rate recovery (HRR) and VO₂ recovery as independent prognostic markers (1). HRR has largely been interpreted as a surrogate of autonomic reactivation, whereas VO₂ recovery has been examined as an indicator of metabolic and hemodynamic restitution (7–11). Despite this physiological interdependence, their combined assessment within a single analytical framework has received limited attention, particularly in stable CAD populations (12).

In this context, some patients with stable CAD may demonstrate preserved heart rate recovery despite delayed VO₂ recovery, suggesting that autonomic and metabolic recovery responses may not always be aligned (13). Such discordance may indicate physiological heterogeneity not fully captured by peak exercise indices (11). These observations highlight the need for an integrated approach to post-exercise recovery assessment rather than reliance on isolated parameters. Accordingly, a phenotype-based approach integrating HRR and VO₂ recovery provides a framework to capture heterogeneity in post-exercise recovery beyond single-parameter assessment. While both HRR and VO₂ recovery have been individually linked to adverse outcomes, their combined assessment remains insufficiently explored in stable CAD populations (14). In addition, CPET-derived variables such as oxygen pulse and ventilatory efficiency may provide complementary insight into cardiovascular performance and recovery physiology. Therefore, the present study aimed to investigate the relationship between HRR and VO₂ recovery and to characterize cardiometabolic recovery patterns, as well as their association with oxygen pulse and ventilatory efficiency.

## Materials and methods

This retrospective observational study was conducted at a tertiary cardiac rehabilitation center between September 2025 and February 2026. The study was performed in accordance with the principles of the Declaration of Helsinki and was approved by the local institutional ethics committee (Approval No: 2026/28; Date: 07/01/2026). Data were obtained from cardiopulmonary exercise testing (CPET) records, and clinical and demographic variables (including age, sex, body mass index, comorbidities, and medication use) were retrieved from the hospital electronic medical record system. As a retrospective analysis of existing data, the requirement for written informed consent was waived by the ethics committee. All CPET measurements were obtained prior to participation in the cardiac rehabilitation program.

Consecutive adult patients (≥18 years) with stable coronary artery disease (CAD) who underwent symptom-limited cardiopulmonary exercise testing during the study period were included. Stable CAD was defined as angiographically documented atherosclerotic coronary artery disease confirmed by invasive coronary angiography or coronary computed tomography angiography, including the presence of coronary plaques and/or prior percutaneous coronary intervention (PCI) or coronary artery bypass grafting (CABG). All patients were clinically stable at the time of testing, with no acute coronary syndrome or coronary revascularization within the preceding 4 weeks.

Patients with atrial fibrillation were excluded due to the inability to reliably assess heart rate recovery. Exercise tests primarily limited by non-cardiac conditions (e.g., musculoskeletal limitations, poor motivation, or technical factors) were excluded. Patients with incomplete first- or second-minute recovery data or technically inadequate CPET recordings were excluded. All CPETs were performed as symptom-limited incremental tests within a real-world pre-rehabilitation setting, with the aim of achieving maximal exercise capacity whenever possible. Although maximal effort was encouraged, achievement of predefined maximal exercise criteria was not required for study inclusion. Exercise was terminated because of limiting symptoms such as dyspnea or fatigue according to standard CPET procedures.

All patients underwent symptom-limited incremental cardiopulmonary exercise testing using a calibrated metabolic cart system according to manufacturer recommendations. Tests were performed on either a treadmill or a cycle ergometer based on clinical indication and standard laboratory practice, using standard incremental protocols. Gas exchange variables were measured breath-by-breath and averaged over 10-s intervals throughout the test, with peak oxygen uptake (VO₂peak) defined as the highest 10-s averaged value achieved during the test.

**Figure 1:**
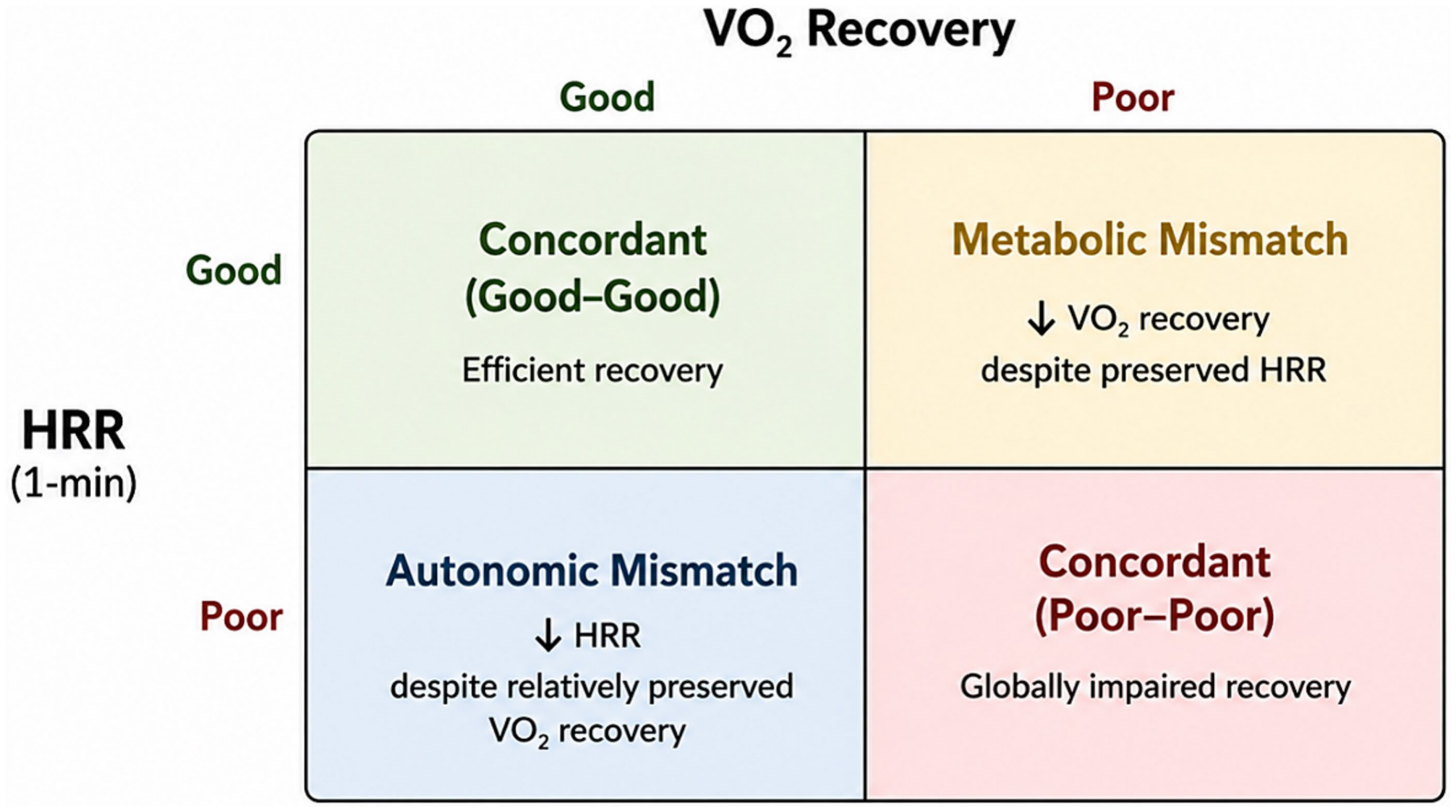
Integrated HRR–VO₂ recovery patterns based on heart rate recovery (HRR) and oxygen uptake (VO₂) recovery. Patients were classified as concordant (good–good or poor–poor) or discordant (metabolic or autonomic mismatch) according to combined recovery patterns. HRR was classified using the established prognostic threshold of HRR1 ≤ 12 bpm, whereas VO₂ recovery was categorized according to the cohort-specific median 1-min VO₂ recovery index.

Following peak exercise, recovery was performed according to a standardized active recovery protocol. For cycle ergometer tests, recovery was conducted at 0 W. For treadmill tests, recovery was performed at a low constant speed with 0% incline. Heart rate and oxygen uptake values during the first and second minutes of active recovery were recorded and used for subsequent analyses (Figure 1).

Heart rate recovery at 1 min (HRR1) was calculated as the difference between peak heart rate and heart rate measured at the first minute of recovery (HRR1 = HR_peak − HR_1min). Impaired HRR was defined as HRR1 ≤ 12 bpm according to established criteria for active recovery protocols (7). Oxygen uptake recovery was defined as the absolute difference between peak oxygen uptake (VO₂peak) and oxygen uptake measured at the first and second minutes of recovery (ΔVO₂1 and ΔVO₂2). In addition, percentage VO₂ recovery relative to VO₂peak was calculated as (ΔVO₂1 / VO₂peak) × 100 and (ΔVO₂2 / VO₂peak) × 100 for subsequent stratification analyses. The absolute ΔVO₂ values reflect the magnitude of oxygen uptake decline during recovery, whereas VO₂ recovery indices provide a normalized measure of recovery relative to the individual peak VO₂ achieved during exercise. All recovery parameters were derived from CPET system recordings and used for subsequent analyses. Patients were categorized into cardiometabolic recovery phenotypes based on combined HRR and VO₂ recovery patterns using a 2 × 2 classification approach. HRR was classified using the established prognostic threshold of HRR1 ≤ 12 bpm, whereas VO₂ recovery was categorized according to the cohort-specific median VO₂ recovery index at 1 min, as no universally accepted clinical cut-off is currently available for VO₂ recovery indices. This study was reported in accordance with the STROBE guidelines.

### Statistical analysis

Statistical analysis was performed using IBM SPSS Statistics (version 26.0, IBM Corp., Armonk, NY, USA). The normality of data distribution was assessed using the Shapiro–Wilk test. Continuous variables were expressed as mean ± standard deviation or median (interquartile range), according to their distribution.

Correlations between heart rate recovery (HRR1) and oxygen uptake recovery parameters (ΔVO₂1, ΔVO₂2, VO₂ recovery indices) were evaluated using Spearman rank correlation analysis due to non-normal data distribution. Pairwise deletion was applied for missing data.

Comparisons across cardiometabolic recovery phenotypes were performed using one-way analysis of variance (ANOVA) for normally distributed variables and the Kruskal–Wallis test for non-normally distributed variables. When an overall group difference was detected, Bonferroni-corrected *post hoc* comparisons were performed as appropriate.

A two-sided *p*-value of <0.05 was considered statistically significant.

## Results

A total of 200 patients were included in the study. Recovery pattern classification was available in 187 patients due to incomplete HRR or VO₂ recovery data. Baseline characteristics of the study population are presented in Table 1.

**Table 1:** Baseline demographic and cardiopulmonary characteristics.

Variable	Value
Age (years)	59.47 ± 9.49
Male sex, *n* (%)	123 (61.5)
BMI (kg/m^2^)	28.25 (IQR: 26.53–32.68)
LVEF (%)	57.25 ± 7.33
Hypertension, *n* (%)	135 (71.1)
Diabetes mellitus, *n* (%)	79 (41.8)
Hyperlipidemia, *n* (%)	128 (67.7)
Beta-blocker use, *n* (%)	121 (61.4)
Medical therapy only, *n* (%)	83 (41.5)
Prior PCI, *n* (%)	91 (45.5)
Prior CABG, *n* (%)	26 (13.0)
VO₂peak (mL/kg/min)	22.50 ± 5.98
VO₂peak (%pred)	90.95 ± 18.54
Peak HR (bpm)	124.90 ± 19.48
Peak HR (%pred)	78.18 ± 11.86
O₂ pulse (mL/beat)	14.94 ± 3.90
VE/VCO₂ slope	32.47 ± 6.34
RERpeak	0.81 ± 0.09
HRR1 (bpm)	18.00 (IQR: 11.00–23.75)
ΔVO₂1 (mL/kg/min)	6.24 ± 3.89
ΔVO₂2 (mL/kg/min)	9.11 ± 4.47
VO₂ recovery index (1 min, %)	27.73 ± 13.14
VO₂ recovery index (2 min, %)	38.92 ± 12.98

Data are presented as mean ± standard deviation for normally distributed variables and median (interquartile range) for non-normally distributed variables.

HRR1 was significantly correlated with ΔVO₂1 (*r* = 0.519, *p* < 0.001) and ΔVO₂2 (*r* = 0.510, *p* < 0.001). Significant correlations were also observed between HRR1 and VO₂ recovery index at 1 min (*r* = 0.496, *p* < 0.001) and 2 min (*r* = 0.531, *p* < 0.001) (Table 2).

**Table 2:** Correlation between HRR1 and oxygen uptake recovery parameters.

Variable	ΔVO₂1	ΔVO₂2	VO₂ recovery index (1 min, %)	VO₂ recovery index (2 min, %)
HRR1 (bpm)	*r* = 0.519, *p* < 0.001	*r* = 0.510, *p* < 0.001	*r* = 0.496, *p* < 0.001	*r* = 0.531, *p* < 0.001

Spearman correlation coefficients are presented.

Table 3 summarizes the significant differences observed across HRR–VO₂ phenotypes for age, VO₂peak, VE/VCO₂ slope, HRR1, and VO₂ recovery parameters. Detailed comparisons across phenotype groups are presented in Table 4. The Good HRR–Good VO₂ group had higher VO₂ recovery indices and ΔVO₂ values, while the Poor HRR–Poor VO₂ group had lower values. Distinct patterns were observed in the discordant phenotypes: the Good HRR–Poor VO₂ group showed lower VO₂ recovery indices, whereas the Poor HRR–Good VO₂ group showed higher VO₂ recovery indices. Similar patterns were consistently observed for ΔVO₂ measures at both 1 and 2 min.

**Table 3:** One-way ANOVA results comparing key variables across HRR–VO₂ phenotypes.

Variable	*F*	df	*p*
Age (years)	12.734	3,196	<0.001
VO₂peak (mL/kg/min)	8.919	3,196	<0.001
VE/VCO₂ slope	4.227	3,196	0.006
HRR1 (bpm)	38.551	3,196	<0.001
ΔVO₂₁	86.744	3,189	<0.001
ΔVO₂₂	34.851	3,183	<0.001
VO₂ recovery index (1 min, %)	131.578	3,189	<0.001
VO₂ recovery index (2 min, %)	46.600	3,183	<0.001

Values represent F statistics from one-way ANOVA. Degrees of freedom are reported as between-groups, within-groups. A *p*-value < 0.05 was considered statistically significant.

**Table 4:** Comparison of cardiopulmonary exercise and recovery parameters across HRR–VO₂ phenotypes.

Variable	Good HRR–Good VO₂ (*n* = 82)	Good HRR–Poor VO₂ (*n* = 33)	Poor HRR–Good VO₂ (*n* = 14)	Poor HRR–Poor VO₂ (*n* = 58)	*p*
LVEF (%)	57.09 ± 7.16	57.12 ± 7.91	52.38 ± 10.56	55.69 ± 8.81	0.096
VO₂peak (mL/kg/min)	25.24 ± 5.54	21.85 ± 4.23	22.44 ± 5.10	19.78 ± 4.52	<0.001
VO₂peak (%pred)	92.64 ± 18.13	95.48 ± 21.02	80.35 ± 23.83	86.85 ± 22.31	0.040
Peak HR (bpm)	131.14 ± 18.98	126.76 ± 20.77	118.65 ± 14.65	115.92 ± 18.79	<0.001
VE/VCO₂ slope	31.89 ± 4.89	33.81 ± 6.06	35.87 ± 6.59	35.58 ± 7.63	0.006
HRR1 (bpm)	22.00 (IQR: 18–27)	18.00 (IQR: 15–23)	8.00 (IQR: 3–11)	6.00 (IQR: 4–10)	<0.001
ΔVO₂1 (mL/kg/min)	9.04 ± 2.90	3.70 ± 1.54	8.96 ± 3.79	2.70 ± 1.80	<0.001
ΔVO₂2 (mL/kg/min)	12.57 ± 4.06	8.00 ± 2.68	10.84 ± 3.99	5.89 ± 3.45	<0.001
VO₂ recovery index (1 min, %)	35.63 ± 7.19	17.17 ± 6.35	39.88 ± 13.91	13.16 ± 7.86	<0.001
VO₂ recovery index (2 min, %)	49.11 ± 9.29	36.68 ± 10.24	48.14 ± 14.60	28.17 ± 12.56	<0.001

Data are presented as mean ± standard deviation or median (interquartile range), according to data distribution. Normally distributed variables were compared using one-way ANOVA, whereas non-normally distributed variables were compared using the Kruskal–Wallis test. A *p*-value <0.05 was considered statistically significant.

*Post hoc* analyses using Tukey HSD revealed that the Good HRR–Good VO₂ group had significantly higher VO₂ recovery values compared to both the Good HRR–Poor VO₂ and Poor HRR–Poor VO₂ groups (both *p* < 0.001). Additionally, the Poor HRR–Good VO₂ group demonstrated significantly higher VO₂ recovery compared to the Poor HRR–Poor VO₂ group (*p* < 0.001). Notably, a significant difference was also observed between the discordant phenotypes, with significantly lower 1-min VO₂ recovery index values in the Good HRR–Poor VO₂ group compared with the Poor HRR–Good VO₂ group (*p* = 0.027).

## Discussion

In this study, heart rate recovery was significantly associated with oxygen uptake recovery following exercise; however, these responses were not consistently aligned across individuals, with a substantial proportion exhibiting discordant recovery patterns. This heterogeneity enabled the identification of distinct cardiometabolic recovery phenotypes, encompassing both concordant and discordant profiles. Together, these findings demonstrate that post-exercise recovery cannot be adequately captured by a single parameter, but rather reflects a multidimensional physiological process.

Heart rate recovery (HRR) is widely recognized as a marker of autonomic function, primarily reflecting parasympathetic reactivation following exercise and consistently associated with cardiovascular outcomes (15). In contrast, oxygen uptake (VO₂) recovery reflects a more integrative physiological process involving cardiovascular, pulmonary, and peripheral metabolic mechanisms related to post-exercise restoration of homeostasis (16). Accordingly, HRR and VO₂ recovery appear to capture complementary rather than interchangeable aspects of recovery physiology, with HRR predominantly reflecting autonomic regulation and VO₂ recovery reflecting the integrated efficiency of oxygen delivery and utilization (16, 17). Although HRR has been shown to correlate with exercise capacity indices such as peak VO₂, this relationship is generally modest and does not fully explain metabolic recovery (15). Despite these physiological differences, HRR and VO₂ recovery have largely been evaluated independently, and their combined clinical interpretation remains insufficiently integrated in clinical practice (18, 19).

In this context, the present study extends existing knowledge by integrating heart rate and oxygen uptake recovery within a unified analytical framework. By jointly evaluating these responses, we identified distinct cardiometabolic recovery phenotypes, including both concordant and discordant patterns, reflecting heterogeneity in post-exercise recovery processes. These findings suggest that recovery after exercise may be governed by partially independent physiological mechanisms rather than a single unified pathway, potentially contributing to inter-individual variability (17–19). From a clinical perspective, this phenotype-based approach may provide a practical framework for pre-rehabilitation assessment and individualized rehabilitation monitoring based on dominant recovery characteristics.

Importantly, *post hoc* analyses revealed a significant difference between the discordant phenotypes, indicating that preserved heart rate recovery does not necessarily correspond to optimal metabolic recovery, and vice versa. This finding suggests that autonomic and metabolic recovery processes may not always be uniformly coupled following exercise. In addition, differences in ventilatory efficiency (VE/VCO₂ slope) across recovery phenotypes further support the potential clinical relevance of this classification. Given that VE/VCO₂ slope is a well-established predictor of mortality and rehospitalization in cardiovascular populations, the observed association between phenotype groups and ventilatory efficiency may indicate underlying differences in clinical risk profiles (9). Consistent with recent evidence indicating that post-exercise recovery may involve partially independent physiological processes (1, 4, 20), we observed discordant recovery patterns in a substantial proportion of patients. Based on the combined evaluation of heart rate and oxygen uptake recovery, four phenotypes emerged within a 2 × 2 framework defined by HRR and VO₂ recovery status. In addition to the concordant profiles, two discordant patterns were identified: metabolic mismatch, characterized by preserved HRR despite impaired VO₂ recovery, and autonomic mismatch, characterized by impaired HRR despite relatively preserved VO₂ recovery. This classification provides a practical framework for characterizing heterogeneous recovery responses beyond single-parameter assessment.

The observed discordant phenotypes likely reflect inter-individual differences in the relative contribution of physiological systems to post-exercise recovery. In this context, impaired oxygen uptake recovery despite preserved HRR may indicate a greater contribution of cardiometabolic limitations affecting oxygen delivery, utilization, or peripheral adaptation. Potential mechanisms may include impaired peripheral oxygen extraction, skeletal muscle deconditioning, or other peripheral limitations affecting recovery kinetics. Conversely, delayed HRR in the presence of relatively preserved VO₂ recovery may reflect alterations in autonomic regulation, reduced vagal reactivation, chronotropic impairment, or medication-related effects despite relatively preserved metabolic recovery. However, the present study was not designed to investigate these mechanisms directly; therefore, these interpretations should be considered hypothesis-generating and require confirmation in prospective mechanistic studies (17, 18).

This interpretation is consistent with evidence demonstrating that CPET-derived parameters reflect integrated cardiovascular, respiratory, and muscular responses and provide complementary rather than interchangeable information (4, 21, 22). Furthermore, the combined evaluation of multiple CPET variables may improve characterization of functional impairment compared with single-parameter assessment (23).

These findings may have important implications for clinical practice, particularly in the context of exercise-based rehabilitation. Contemporary evidence supports the use of CPET to guide individualized rehabilitation strategies through comprehensive evaluation of functional capacity and underlying physiological limitations (22–25). In this context, the proposed recovery classification framework may enhance pre-rehabilitation assessment by incorporating recovery dynamics. Specifically, impaired VO₂ recovery despite preserved HRR may indicate the need for closer monitoring of metabolic tolerance during rehabilitation, whereas delayed HRR in the presence of relatively preserved VO₂ recovery may warrant greater attention to autonomic responses during exercise training (26). However, further prospective studies are needed to determine the clinical applicability and prognostic significance of these recovery patterns (27).

This study has several limitations that should be considered. First, the retrospective and cross-sectional design precludes causal inference between heart rate recovery and oxygen uptake recovery responses. Second, the study was conducted at a single center and included only patients with coronary artery disease, which may limit generalizability to other clinical populations. Nevertheless, this relatively homogeneous cohort may have allowed a more focused evaluation of recovery dynamics within a specific cardiovascular context. Third, potential selection bias cannot be excluded, as only patients who underwent CPET were included in the analysis. In addition, recovery parameters were assessed at predefined time points and may not fully capture the complexity of recovery kinetics. Detailed information regarding beta-blocker dosage and other chronotropic medications was not consistently available due to the retrospective design and therefore could not be evaluated. Finally, the proposed recovery classification should be considered exploratory and hypothesis-generating until externally validated in prospective studies.

Heart rate recovery and oxygen uptake recovery were significantly associated, yet not consistently aligned across individuals, revealing distinct cardiometabolic recovery phenotypes. These findings indicate that post-exercise recovery is a multidimensional process that cannot be adequately characterized by a single parameter alone. By integrating HRR and VO₂ recovery within a unified framework, the present study proposes an integrated recovery classification framework that reflects heterogeneity in recovery dynamics and may support pre-rehabilitation assessment in patients with coronary artery disease. The observed association between recovery phenotypes and ventilatory efficiency (VE/VCO₂ slope) further supports the potential clinical relevance of this classification and its possible role in individualized rehabilitation monitoring strategies.

## Data Availability

The raw data supporting the conclusions of this article will be made available by the authors, without undue reservation.
